# Dexmedetomidine as a total intravenous anesthetic in pediatric patients undergoing cleft lip and palate surgery: a case series

**DOI:** 10.1186/s13256-024-04645-6

**Published:** 2024-07-16

**Authors:** Corry Quando Yahya, Lucky Andriyanto, Yantoko Azis Priyadi

**Affiliations:** 1 Department of Anesthesiology and Critical Care Medicine, Faculty of Medicine, Universitas Airlangga, Jl. Mayjen Prof. Dr. Moestopo No. 6-8, Airlangga, Gubeng, Surabaya, 60286 Indonesia; 2 Department of Plastic and Reconstructive Surgery, Rumah Sakit Umum Pusat Persahabatan, Jl. Persahabatan Raya No. 1, Jakarta Timur, 13230 Indonesia

**Keywords:** Anesthesia, Case report, Case series, Cleft lip, Cleft palate, Dexmedetomidine, Total intravenous

## Abstract

**Background:**

Surgery for pediatric cleft lip and palate repair often utilizes high-dose opioids and inhaled anesthesia, thereby causing postoperative complications such as desaturation and/or severe agitation after anesthesia. These complications are detrimental to the child and medical personnel and cause tremendous psychologic stress to parents. Our aim is to decrease these complications through dexmedetomidine, an alpha-2 receptor agonist with anxiolytic, sympatholytic, and analgetic properties. Devoid of respiratory depressant effect, it allows patients to maintain effective ventilation and reduce agitation, postoperatively. Its unique anesthetic property may shed light on providing safe anesthesia and gentle emergence to this young, vulnerable population.

**Case presentation:**

A total of 21 patients of Sundanese ethnicity, aged 3 months to 8 years (9 males and 12 females), underwent cleft lip or cleft palate surgery using total intravenous dexmedetomidine. Anesthesia was induced using sevoflurane, fentanyl, and propofol, and airway was secured. Intravenous dexmedetomidine 1.5 μg/kg was administered within 10 minutes, and a maintenance dose of 1.5 μg/kg/hour was continued as the sole anesthetic maintenance agent thereafter. Hemodynamics and anesthetic depth using Patient State Index (SEDLine™ monitor, Masimo Corporation, Irvine, CA, USA) were monitored carefully throughout the surgical procedure. Dexmedetomidine did not cause any hemodynamic derangements or postoperative complications in any of our patients. We found agitation in 9.5% (2/21) of patients.

**Conclusion:**

Dexmedetomidine can be used as a total intravenous anesthetic agent to maintain anesthesia and provide gentle emergence to infants and young children undergoing cleft lip and palate repair.

## Background

Cleft lip and cleft palate constitutes one of the most common craniofacial congenital anomalies found in the Asian population with an incidence of 1.5 for every 1000 births [1]. Pediatric patients with these defects will require early repair to prevent development delay as a result of feeding difficulties, speech impairment, and deafness due to recurrent chronic ear infections. As a result, the recommended optimal time for repair in these children is 3 months of age for labioplasty and 6 months of age for palatoplasty [2]. However, anesthesia for this special population poses its own risks and challenges involving the airway [3].

Studies have found almost 50% of patients with cleft lip and palate repair to acquire some form of syndrome. In the absence of a syndrome, difficult laryngoscopy was reported at 7.06% in babies 1–6 months of age, 2.9% in 6–12 months, and 3.13% in children 1–3 years of age [4]. An audit involving 1000 pediatric cleft lip and palate surgeries under the Smile Train project found intraoperative complications to occur in 2.4% of cleft lip cases and 8.7% of cleft palate cases, with an overall mortality of 0.2% attributable to postoperative hypoxia [5]. In fact, the majority of morbidity cases were due to airway problems, which includes difficult intubation, inadvertent extubation during the surgical procedure, and airway obstruction during the recovery period [6, 7].

General anesthesia is the common preferred technique in facilitating cleft repair in the pediatric population. As a result, high-dose opioids and inhalation anesthetics are given as a routine anesthetic regimen. Not surprisingly, the majority of these patients experience emergence agitation (EA) upon recovery from anesthesia [8]. The incidence of EA varies from 10% to 80% and poses danger for both patient and caregivers [9]. Maladaptive behaviors such as hysterical screaming, kicking, and thrashing may cause postoperative complications such as tongue edema, rebleeding from surgical sites, inadvertent intravenous catheter removal, bronchospasm, and wound dehiscence, all of which prolong hospital stays and may produce a sentinel event [10].

Until today, there is no comparative study between different anesthetic agents or a consensus on the safest anesthetic agent to be used for pediatric patients undergoing cleft lip and cleft palate repair. An ideal anesthetic agent would preferably possess anxiolytic, sympatholytic, and analgetic properties without residual respiratory depressant effects or agitation after its application.

These properties are exhibited by dexmedetomidine, a selective alpha-2 receptor agonist with an α2:α1 receptor ratio of 1620:1 [11, 12]. Contrary to propofol and benzodiazepines, dexmedetomidine’s sedative effect does not stem from the gamma-aminobutyric acid (GABA) receptor [13]. Its sedative effects are mediated through the activation of central alpha-2 presynaptic and postsynaptic receptors in the locus coeruleus, thereby mimicking a state of deep sleep [14–16]. Meanwhile, its anxiolytic, sympatholytic, and analgesic effects are mediated through the activation of postsynaptic adrenergic receptors located in the vasomotor medullary center in the brain and spinal cord. The activation of these receptors prevents the release of norepinephrine and reduces neuronal firing, hence minimizing nociceptive and pain signals. As sympathetic tone is reduced, heart rate and blood pressure are also effectively reduced [17].

Dexmedetomidine has been studied since 1999, and its use as a sedative agent for diagnostic procedures in intensive care units, as an adjuvant anesthetic, and even in painful procedures such as laryngoscopy has been well documented [16, 18–22]. In fact, the majority of its studies were focused on the pediatric population [23]. Dexmedetomidine’s unique features are not comparable to other anesthetic drugs available. Its ability to produce analgesia and hypnosis without depressing respiration or inciting agitation is a major advantage to pediatric patients. Herein, we aim to report its safety and efficacy in pediatric patients undergoing cleft lip and cleft palate repair.

## Case presentation

We identified 23 Sundanese patients between the ages of 3 months and 10 years who were eligible for cleft lip or cleft palate surgery: 11 were male, and 12 were female. All the children were examined the day before surgery, and written informed consent was obtained from each parent. Patients with a respiratory tract infection, with a potentially difficult airway examined through the COPUR index, who were syndromic, who were severely comorbid, or who had an American Society of Anesthesiologist (ASA) physical status classification ≥ 3 were excluded from this event.

All the children were fasted for 6 hours for dairy products and solid food, 4 hours for breastmilk, and 2 hours for water. No premedication was administered. The child was taken to the operating theater (OT) and induced with 100% oxygen and 8% sevoflurane to facilitate an intravenous line placement followed by a bolus injection of 2 µg/kg fentanyl and 3 mg/kg of propofol. Laryngoscopy for endotracheal tube insertion or laryngeal mask insertion was performed gently to avoid bucking and inadvertent laryngospasm, using the appropriately sized laryngeal mask or nonkinking endotracheal tube.

After securing the airway, sevoflurane inhalation was promptly stopped and all children were observed breathing spontaneously via a Mapleson D system attached to the endotracheal tube or laryngeal mask on 100% oxygen and flow rate of 4–6 liters per minute. A loading dose of intravenous dexmedetomidine at 1.5 μg/kg was administered within 10 minutes, followed by a maintenance dose of 1.5 μg/kg/hour using a syringe pump.

Routine noninvasive monitoring included parameters such as heart rate (HR), systolic blood pressure, diastolic blood pressure, mean arterial pressure (MAP), and oxygen saturation (SpO2). Root Sedline Monitor was used to perform processed electroencephalography (pEEG) by placing sensors on the child’s forehead and ensure adequate anesthetic depth during surgery.

**Fig. 1:**
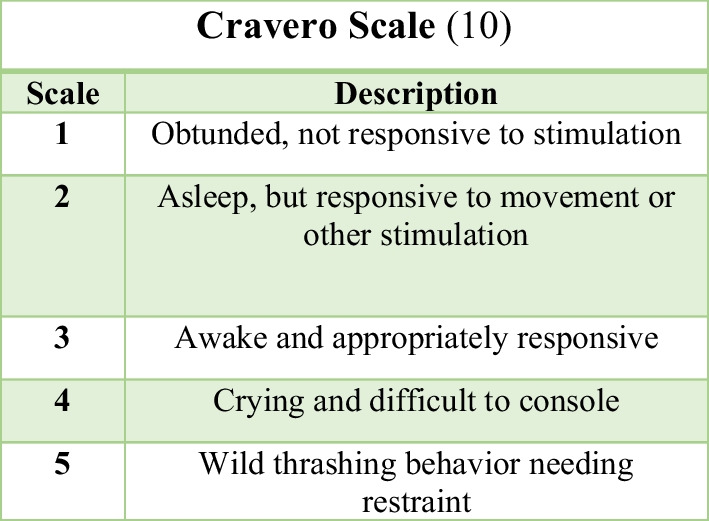
Cravero agitation scale

Before incision, local anesthetic infiltration using 2% lidocaine HCl and 1: 80,000 epinephrine (Pehacaine® 2%) was given. If there were any sign of awareness, as presented by a rising Patient State Index (PSI) value above 50 or the presence of any spontaneous movement of the extremities, rescue propofol at a dose of 3 mg/kg was given as an intravenous bolus. Fifteen minutes before the end of surgery, patients were given 15 mg/kg of intravenous paracetamol for postoperative analgesia. At the end of surgery, Dexmedetomidine (DEX) infusion was discontinued. Time to extubation, time to awareness, postoperative agitation using the Cravero scale (Fig. 1), and postoperative complications such as desaturation and laryngospasm were carefully observed.

**Fig. 2:**
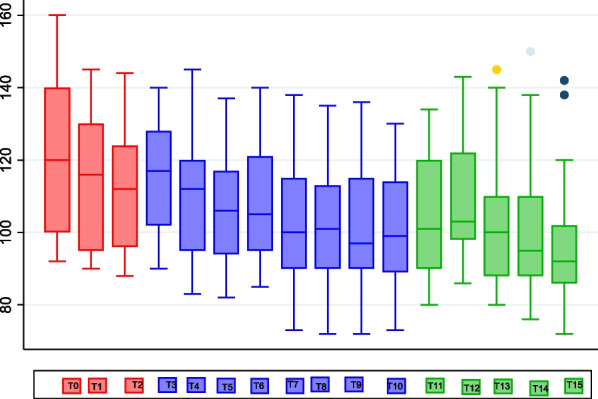
Changes in heart rate during different time frames. Data are expressed as mean ± standard deviation (SD). T0, preinduction; T1, preintubation; T2, post-intubation and start of dexmedetomidine loading dose; T3, lidocaine infiltration; T4–10, start of surgery and recorded 10 minutes thereafter; T11, end of operation; T12, extubation; T13–15, 0.5, 1, and 2 hours in the recovery room

Twenty-three patients were scheduled for lip or cleft surgery using intravenous dexmedetomidine. However, two of these patients (two male children: 2 years and 8 years) did not receive dexmedetomidine as their sole anesthetic maintenance agent and, hence, were excluded from the result reports. Throughout the procedure, there were no incident of hypertension, hypotension, or bradycardia with dexmedetomidine loading dose or maintenance dose. Changes in heart rate before, during, and after surgery are shown in Fig. 2.

One male patient suffered recurrent episodes of bronchospasm that resolved only after sevoflurane administration despite an adequate PSI value of 25–35, while another male child (8 years old) received 0.5% sevoflurane and dexmedetomidine infusion to maintain a PSI value of 25–35. Clinical characteristics, demographic data, duration, and type of surgery are listed in Table 1. Ten pediatric patients (48%) underwent labioplasty, and 11 patients (52%) underwent palatoplasty. The duration of anesthesia ranged from around 37 minutes for labioplasty to 54 minutes for palatoplasty, while the duration of surgery ranged from 28 minutes for labioplasty to 39 minutes for palatoplasty. The average total dose of dexmedetomidine administered to labioplasty patients was 27 µg, while palatoplasty patients received 30 µg.

**Table 1:** Descriptive statistics of patient by gender, age, weight, and type of surgery

	Labioplasty (*n* = 10)	Palatoplasty (*n* = 11)
Age (months)	43 ± 33 (3–84)	28 ± 22 (11–84)
Gender (M/F)	3/7	6/5
Weight (kg)	12.6 ± 6.1 (5–23)	12.2 ± 3.9 (8–21.5)
Anesthesia duration (minutes)	37 ± 10 (15–57)	54 ± 16 (25–90)
Surgical duration (minutes)	28 ± 7 (10–35)	39 ± 12 (20–60)

Data are expressed as mean ± standard deviation (minimum – maximum)

Administration of dexmedetomidine for labioplasty and palatoplasty surgery did not affect extubation time. Anesthetic maintenance with dexmedetomidine for 39 minutes (labioplasty) versus 52 minutes (palatoplasty) produced the same outcome: extubation time of 9 minutes; awakening time of 41 minutes for labioplasty patients and 46 minutes for palatoplasty patients (Table 2).

**Table 2:** Anesthesia characteristics

	Labioplasty (*n* = 10)	Palatoplasty (*n* = 11)
Anesthesia duration (minutes)	37 ± 10 (15–57)	54 ± 16 (25–90)
Total DEX used ($\upmu$g) (Loading and maintenance dose)	27 ± 16 (7–61)	30 ± 7 (23–48)
Time to extubation (minutes)	9 ± 5 (1–16)	9 ± 5 (2–17)
Time to awakening (minutes)	43 ± 13 (25–60)	45 ± 7 (34–60)

Data are expressed as mean ± standard deviation (min – max)

*DEX* Dexmedetomidine

We did not find any ventilation or intubation difficulties in this report. However, two children experienced laryngospasm and desaturation without bradycardia during anesthesia induction. After surgery, each child was monitored in the recovery room for their hemodynamic status and agitation scale. Agitation scale was evaluated every 15 minutes (Table 3). In this report, we found two patients who were agitated with a mean Cravero scale of 4.3 and mean duration of 60 minutes (60–120 minutes). Those who were not agitated had a mean Cravero scale of 2.3–3.5 from admission to the recovery room until their awakening time.

**Table 3:** Cravero agitation scale after intravenous dexmedetomidine

Time interval	Agitation scale	
	Labioplasty	Palatoplasty
0 minutes	2.3 (1.81–2.78)	2.45 (1.69–3.21)
15 minutes	2.3 (1.81–2.78)	2.27 (1.66–2.88)
30 minutes	2.4 (1.89–2.90)	2.45 (1.75–3.15)
45 minutes	2.8 (2.34–3.25)	3.27 (2.66–3.88)
60 minutes	3.2 (2.89–3.50)	3.36 (2.82–3.90)

Data are expressed as mean and interquartile range (IQR)

## Discussion

Dexmedetomidine is a selective alpha 2-adrenoceptor agonist that exhibits a wide variety of effects due to its widespread distribution within the body [18]. This drug has been introduced since 1999, and its effect on the pediatric population has been thoroughly studied. DEX can be used as a sedative agent in the intensive care setting, for procedural sedation, as an adjuvant, and even as a main anesthetic agent to babies and children undergoing surgical procedures [23]. The literature recommends an intravenous loading dose of 1 μg/kg within 10 minutes, followed by a maintenance dose of 0.7 μg/kg/hour. This would yield an average dexmedetomidine plasma concentration of 600 pg/mL, the dose to provide adequate sedation [14].

In this report, we found using the recommended dosing frequently produced an average PSI value above 50 within 5–8 minutes after anesthesia induction. The delay in reaching adequate PSI value may be caused by dexmedetomidine’s onset of 15 minutes. The pediatrics age group of 2 months to 6 years has a faster clearance time (0.8–1.2 L/kg/hour) as compared with older children and adults (0.6–0.7 L/kg/hour) [13]. Furthermore, in numerous reports, dexmedetomidine was used as an adjuvant anesthetic, not provided at maintenance doses. In this report, dexmedetomidine was the sole anesthetic agent and may therefore need a higher loading dose to quickly achieve the concentration needed for anesthesia and keep patients under hypnosis. Hence, we revised a loading dose of 1.5 μg/kg administered within 10 minutes followed by a maintenance dose of 1.5 μg/kg/hour.

With this dose, we were able to provide adequate sedation to infants and children aged 3 months to 8 years old (weight 5–23 kg) and achieve a stable PSI value ranging from 23 to 38. However, we discovered that children weighing above 23 kg needed another anesthetic agent to achieve a PSI value of 25–50. Hence, we combined the use of sevoflurane 0.5 vol% and intravenous dexmedetomidine for our 8-year-old, 30 kg patient for cleft repair.

Bradycardia is the most common side effect reported in dexmedetomidine but seldom requires any pharmacologic intervention [15]. In this report, we did not encounter any hemodynamic derangements after administration of dexmedetomidine loading dose or maintenance dose.

Evaluation of anesthetic depth with PSI values is not a dynamic real-time monitoring as the equipment has a“lag-time.” This means that spontaneous movements may occur even before changes in PSI values are able to warn the anesthetist of possible awareness. With this in mind, we recommend the administration of rescue propofol before incision or infiltration of local anesthetics to maintain an adequate anesthetic depth while awaiting dexmedetomidine to reach its onset. With the help of pEEG monitoring, some important learning points can be derived:

**Table:** 

Advantages	Disadvantages
(1) PSI value of 25–50 can be attained using dexmedetomidine alone in children weighing less than 23 kg	(1) PSI value of 25–50 cannot be attained with dexmedetomidine alone in children weighing more than 23 kg. Another adjuvant is necessary to provide adequate anesthesia
(2) Does not cause unwanted side effects such as bradycardia, hypertension, or hypotension	(2) Dexmedetomidine requires 15–20 minutes to reach its peak effect
(3) Cost effective without toxic waste or end products	(3) Required longer awakening time (41–50 minutes) compared with sevoflurane inhalation anesthesia (24–46 minutes)
(4) Reduced incidence of postoperative agitation	
(5) Lightweight and relatively simple to prepare in a resource-poor facility. Dexmedetomidine 200 μg per 2-ml vial is prepared using a syringe, extension tubing, three-way extension, isotonic solution, and syringe pump	

*PSI* patient state index

Upon emerging from anesthesia, the majority of the children reacted to stimulation by wrinkling their forehead, giving a brief cry, and moving their extremities toward the stimulation site. When the stimulation was stopped, the children returned to their sleeping state without any incident of apnea, hypopnea, laryngospasm, or desaturation in the recovery room. Awakening time after receiving dexmedetomidine was indeed longer (45 ± 7 min) compared with sevoflurane (35 ± 11 min) [24]. Nevertheless, we found a profoundly calm child gently emerging from anesthesia as the effects of dexmedetomidine wore off. The child awakened fully conscious, able to identify caregivers and display appropriate emotional reaction, all of which are a sight of rare occurrence in pediatric anesthesia.

## Conclusion

Despite its limitation as a sole intravenous anesthetic agent in all pediatric age groups, we found dexmedetomidine to be safe and effective in younger pediatric patients (< 8 years old) undergoing cleft lip and cleft palate surgery. Its unique characteristics keep the airway tone intact, preserve ventilatory drive, and reduce postoperative agitation, all of which are beneficial, especially in pediatric anesthesia involving the airway. Lastly, emergence from anesthesia can be a gentle and tranquil event, thereby providing better parent satisfaction and reducing the risk of sentinel events and prolonged hospital stay.

## Data Availability

Please contact author for data requests.
